# No Threshold Exists for Recommending Revision Surgery in Metal-on-Metal Hip Arthroplasty Patients With Adverse Reactions to Metal Debris: A Retrospective Cohort Study of 346 Revisions

**DOI:** 10.1016/j.arth.2019.03.022

**Published:** 2019-07

**Authors:** Gulraj S. Matharu, Fiona Berryman, David J. Dunlop, Matthew P. Revell, Andrew Judge, David W. Murray, Hemant G. Pandit

**Affiliations:** a Nuffield Department of Orthopaedics, Rheumatology and Musculoskeletal Sciences, University of Oxford, Nuffield Orthopaedic Centre, Oxford, United Kingdom; b Musculoskeletal Research Unit, Bristol Medical School, University of Bristol, Bristol, United Kingdom; c Research Department, The Royal Orthopaedic Hospital, Birmingham, United Kingdom; d Leeds Institute of Rheumatic and Musculoskeletal Medicine, Chapel Allerton Hospital, University of Leeds, Leeds, United Kingdom

**Keywords:** adverse reactions to metal debris, metal-on-metal hip arthroplasty, outcomes, predictors, revision surgery

## Abstract

**Background:**

Surgeons currently have difficulty when managing metal-on-metal hip arthroplasty (MoMHA) patients with adverse reactions to metal debris (ARMD). This stems from a lack of evidence, which is emphasized by the variability in the recommendations proposed by different worldwide regulatory authorities for considering MoMHA revision surgery. We investigated predictors of poor outcomes following MoMHA revision surgery performed for ARMD to help inform the revision threshold and type of reconstruction.

**Methods:**

We retrospectively studied 346 MoMHA revisions for ARMD performed at 2 European centers. Preoperative (metal ions/imaging) and intraoperative (findings, components removed/implanted) factors were used to predict poor outcomes. Poor outcomes were postoperative complications (including re-revision), 90-day mortality, and poor Oxford Hip Score.

**Results:**

Poor outcomes occurred in 38.5%. Shorter time (under 4 years) to revision surgery was the only preoperative predictor of poor outcomes (odds ratio [OR] = 2.12, confidence interval [CI] = 1.00-4.46). Prerevision metal ions and imaging did not influence outcomes. Single-component revisions (vs all-component revisions) increased the risk of poor outcomes (OR = 2.99, CI = 1.50-5.97). Intraoperative modifiable factors reducing the risk of poor outcomes included the posterior approach (OR = 0.22, CI = 0.10-0.49), revision head sizes ≥36 mm (vs <36 mm: OR = 0.37, CI = 0.18-0.77), ceramic-on-polyethylene revision bearings (OR vs ceramic-on-ceramic = 0.30, CI = 0.14-0.66), and metal-on-polyethylene revision bearings (OR vs ceramic-on-ceramic = 0.37, CI = 0.17-0.83).

**Conclusion:**

No threshold exists for recommending revision in MoMHA patients with ARMD. However postrevision outcomes were surgeon modifiable. Optimal outcomes may be achieved if surgeons use the posterior approach, revise all MoMHA components, and use ≥36 mm ceramic-on-polyethylene or metal-on-polyethylene articulations.

Metal-on-metal hip arthroplasty (MoMHA) has been associated with high implant failure rates [1], [2], with adverse reactions to metal debris (ARMD) representing the commonest indication for revision [3], [4]. Despite these poor outcomes of primary MoMHAs, approximately 80% of these implants remain in-situ worldwide [5], [6]. Given the prevalence of ARMD revision surgery is increasing [3], [4], [5], [6], [7], many more MoMHA patients are likely to undergo future revision.

The frequency of complications (≤68%) and re-revisions (≤38%) following ARMD revision have been variable [8]; however, the poor short-term outcomes initially reported [9], [10] have seemingly improved in recent studies [11], [12]. This most likely reflects more regular MoMHA patient surveillance and surgeons lowering their threshold for revision given the initial poor outcomes of revisions [9], [13], [14], [15]. However, little good evidence exists regarding outcomes following ARMD revision, with a recent systematic review highlighting that studies generally involved small (<100 patients) single-center cohorts with short-term follow-up (mean 3 years) [8]. Surgeons therefore will struggle to informatively counsel MoMHA patients about the risks associated with ARMD revision surgery. Furthermore, surgeons currently have no robust information regarding the threshold (when to recommend revision) and type of surgery (what reconstruction to perform) required in MoMHA patients with ARMD. This stems from a lack of evidence, which is emphasized by the variability in the recommendations proposed by different worldwide regulatory authorities for considering revision [16]. Although some studies have identified factors predicting poor outcomes following ARMD revision, including solid pseudotumors, these have been small and underpowered [7], [15], [17], [18]. Therefore, surgeons currently have difficulty when managing MoMHA patients with ARMD.

Identifying any prognostic factors of outcome following ARMD revision would assist surgeons when making decisions regarding the threshold and type of reconstruction to perform, with this information also helpful when counseling patients before and after revision regarding their likely outcomes. Establishing a robust threshold for recommending ARMD revision surgery is critical, not only so patients can have surgery at the correct time and obtain the best possible outcomes but also to prevent individuals being exposed to unnecessary revisions. A recent registry analysis of 2535 MoMHAs revised for ARMD identified factors predictive of re-revision that were surgeon modifiable, including the revision articulation [19]. Although these findings provide surgeons with useful information when planning reconstructions for ARMD, registries do not collect data on important prerevision factors, such as blood metal ions and cross-sectional imaging, which are crucial for establishing a threshold for MoMHA revision surgery.

We performed a large retrospective cohort study involving MoMHA patients undergoing revision surgery for ARMD. We aimed to determine the outcomes following ARMD revision and identify predictors of a poor outcome. The latter predictors would be used to inform the threshold for ARMD revision and the type of reconstruction.

## Materials and Methods

### Study Design, Selection Criteria, and Definitions

We performed a retrospective cohort study involving 2 specialist European arthroplasty centers. The study included all patients with MoMHAs undergoing revision surgery for ARMD between January 2001 and March 2016. Cases were identified from prospectively maintained institutional databases described previously [3], [17], [20], [21]. This study was registered with each institution’s review board, with all patients reviewed according to institutional follow-up protocols.

During the study period, 706 MoMHA revisions were performed for any indication at the 2 centers. There were 346 (49.0%) revisions performed for confirmed ARMD, which were included in this study. Both centers were tertiary units involving 16 surgeons. Although all ARMD revisions were performed at 2 centers, the index MoMHA surgery could have been performed elsewhere. In addition to primary MoMHAs requiring revision for ARMD, we also included primary MoMHAs revised to another MoMHA for non-ARMD indications (eg, hip resurfacing with femoral neck fracture revised to a stemmed MoMHA), which subsequently required revision for ARMD.

Preoperative investigations and intraoperative and histopathological findings were all used for diagnosing ARMD. Features of ARMD identified on preoperative cross-sectional imaging and/or intraoperatively included metallosis, pseudotumor, synovitis, joint effusion, tissue damage, and/or necrosis [12], [17], [22]. A pseudotumor was defined as a cystic, solid, or mixed mass communicating with the hip joint [7], [12], [23]. Histological evidence supportive of ARMD included lymphocytic infiltrates (including aseptic lymphocytic vasculitis and associated lesions) and/or phagocytic macrophage responses to metal wear debris, with or without tissue necrosis [24], [25], [26].

### Preoperative Investigations

Patients with problematic MoMHAs attended outpatient clinics for assessment because of one of the following: (1) patients were symptomatic and were seen either during or in-between scheduled reviews; (2) patients were discharged but subsequently referred back by the general practitioner because of new symptoms; (3) patients were asymptomatic and under surveillance recommended by regulatory authorities [13], [14], [27] with abnormalities identified during these investigations; and (4) symptomatic or asymptomatic patients with abnormal investigations were referred from another center for specialist management.

The routine preoperative investigation of patients with problematic MoMHAs has been described in detail, including the methods for blood metal ion sampling and cross-sectional imaging [3], [20], [21]. All patients underwent clinical examination and radiographic assessment with standardized anteroposterior pelvic radiographs, with or without a lateral hip radiograph. Most patients also underwent blood metal ion sampling (cobalt and chromium concentrations) and cross-sectional imaging (ultrasound and/or metal artifact reduction sequence magnetic resonance imaging). However, these investigations were not performed prior to some of the earliest revisions given that the understanding of ARMD evolved gradually with time [9], [15]. All blood metal ion samples were analyzed at accredited laboratories, and all cross-sectional imaging was performed and interpreted by expert musculoskeletal radiologists.

### Revision Surgery and Follow-Up

The decision to perform revision was made by the patient’s surgeon based on symptoms and/or investigative findings. The indications for revision have evolved over time [15], [28]. The earliest revisions were performed in symptomatic patients with large and sometimes destructive ARMD lesions. However, as outcomes following these early revisions were poor [9], [15], the indications for ARMD revision surgery were broadened to include mildly symptomatic patients with less severe disease. In general, all diseased tissue (inflamed/necrotic), including pseudotumors, were excised completely, though this was not possible in all cases because of proximity to neurovascular structures. The specific reconstruction performed for ARMD (which components were removed, the design, fixation, head size and bearing surface of the new implants, and the use of bone graft) was at the discretion of the operating surgeon. Details of routine postoperative care after revision has been described [17], [29].

After revision, patients were reviewed in clinic at 6 weeks and 1 year postoperatively. Subsequent reviews were according to clinical need, usually annually. Consultations included clinical examination, radiographs (anteroposterior pelvis with or without lateral hip), and completion of the Oxford Hip Score (OHS) questionnaire [30], [31]. Patients with pain after revision underwent further investigation as required, including blood tests (to assess for infection and further metal ion release), cross-sectional imaging, and joint aspiration.

### Data Collection and Outcomes of Interest

Relevant preoperative and intraoperative factors and postrevision outcomes were collected using standardized data collection proformas described previously [17], [28]. All data were obtained retrospectively from the clinical notes, the electronic patient records systems, and the prospectively maintained institutional databases.

Preoperative variables included age, gender, details of the MoMHA (including manufacturer), unilateral/bilateral MoMHA, local and systemic symptoms, radiographic findings, blood cobalt and chromium concentrations, and cross-sectional imaging abnormalities (including the volume and consistency of any lesions identified). Radiographic acetabular component position (inclination and version) was determined using validated methods with ImageJ (National Institutes of Health, Bethesda, MD) [32]. Radiographs were assessed by 2 reviewers blinded to the clinical information, with 50 radiographs assessed by both reviewers. Intraclass correlation coefficients between observers were excellent: inclination = 0.979 (95% confidence interval [CI] = 0.955-0.990), version = 0.968 (95% CI = 0.947-0.988). Acetabular components were considered malpositioned if one or both parameters were outside the recommended optimal zone for MoMHAs (inclination 35°-55° and anteversion 10°-30°) [33]. Each radiograph was systematically analyzed for evidence of implant failure as described previously, including component loosening, osteolysis, and heterotopic ossification [34].

Intraoperative variables were identified from the lead surgeon’s operation records, which were assessed by 1 independent observer who was not involved with the surgeries and blinded to the preoperative investigation findings. Data extracted included details of the surgeon, approach, intraoperative findings (including pseudotumor, effusions, soft-tissue damage, osteolysis, metallosis, synovitis, fracture, infection, necrosis, and component position), components removed (all, single-component, or modular components), and the reconstruction performed.

The study outcomes of interest following ARMD revision were as follows: (1) intraoperative complications; (2) postoperative complications; (3) re-revision surgery, (4) further surgery excluding re-revision; (5) patient-reported outcome measures (PROMs), and (6) mortality. Re-revision surgery was defined as removal, exchange, or addition of any implant. If further surgery was performed elsewhere, the respective hospital was contacted to complete data collection (date, indication, and procedure performed). Both centers used the OHS (0-48; 48 = best outcome) for postoperative PROMs. We defined a poor PROM as an OHS under 27 points, as recommended previously [31], [35]. Patients who had not been reviewed within 12 months were sent a postal PROM and further surgery questionnaire to complete. All deaths were investigated using patient notes and information held by the general practitioner to determine whether the death was related to revision arthroplasty and whether the hip was re-revised or remained in-situ at the time of death.

### Statistical Analysis

All statistical analysis was performed using Stata, version 14.2, (StataCorp., College Station, TX). The significance level was *P* < .05, with 95% CIs also used. For numerical data, either the median and interquartile range or the mean and standard deviation or range were used depending on the data distribution. Implant survival analysis was performed using the Kaplan-Meier method with re-revision surgery used as the end point. Patients not undergoing re-revision were censored at latest follow-up (clinic review, questionnaire completion, or death).

Logistic regression modeling was used to identify predictors of a poor outcome. A poor outcome was defined as one or more of the following: intraoperative complication, postoperative complication, further surgery or procedure (including re-revision), mortality within 90 days of surgery, and poor PROMs. Regression models were based on a subgroup of patients who all had blood metal ions and cross-sectional imaging before ARMD revision. Univariable models explored the association between each predictor and poor outcome. For continuous predictors, linearity was assessed using fractional polynomials, with data categorized if the relationship between the predictor and outcome was nonlinear. Multivariable logistic regression models were devised based on (1) preoperative factors only (threshold for surgery) and (2) intraoperative factors only (reconstruction required). These 2 multivariable models were developed using stepwise selection methods, with the area under the curve (AUC) calculated to assess the discriminatory performance of each model (50% AUC = nondiscriminatory and 100% AUC = perfect discrimination). The *P* values for the removal and inclusion of predictors in the final multivariable models were *P* ≥ .20 and *P* < .10, respectively. Regression diagnostics were assessed to ensure all underlying model assumptions were met [36], [37]. When developing predictive models, it has been recommended that to ensure sufficient power, there should be at least 10 outcome events per candidate variable (ie predictor) assessed, although there is also evidence that fewer than 10 outcome events per predictor can be satisfactory [38].

## Results

There were 346 MoMHAs revised for ARMD that were eligible for inclusion, with the respective preoperative and intraoperative factors summarized (Table 1).

**Table 1:** Preoperative and Intraoperative Factors Affecting Outcomes Following ARMD Revision Surgery.

Factor	Whole Cohort (n = 346)	Cohort With Ions & Imaging (n = 239)	Good Outcome (n = 147; 61.5%)	Poor Outcome (n = 92; 38.5%)	Univariate LR Odds Ratio (95% CI)	*P* Value
**Preoperative factors**						
Mean age at revision in years (SD)	59.7 (10.8)	59.9 (10.6)	61.0 (9.9)	58.3 (11.5)	0.98 (0.95-1.01)	.061
Female gender (%)	231 (66.8)	168 (70.3)	106 (72.1)	62 (67.4)	0.80 (0.45-1.41)	.438
Bilateral MoM hips any (%)	118 (34.1)	93 (38.9)	60 (40.8)	33 (35.9)	0.81 (0.47-1.39)	.446
Bilateral MoM hips revised for ARMD (%)	46 (13.3)	35 (14.6)	25 (17.0)	10 (10.9)	0.60 (0.27-1.30)	.195
Mean time to revision for ARMD (SD)	6.7 (3.3)	7.1 (3.2)	7.4 (3.2)	6.6 (3.3)	0.92 (0.85-1.01)	.051
Time to revision under 4 y (%)	72 (20.8)	37 (15.5)	17 (11.6)	20 (21.7)	2.12 (1.05-4.31)	**.037**
Primary and revision center same (%)	249 (72.0)	182 (76.2)	110 (74.8)	72 (78.3)	1.21 (0.65-2.25)	.545
Primary revision indication (%)						
Primary osteoarthritis	267 (77.2)	180 (75.3)	117 (79.6)	63 (68.5)	1.00 (ref)	
Other diagnoses (native hip)	64 (18.5)	47 (19.7)	24 (16.3)	23 (25.0)	1.78 (0.93-3.40)	.082
Failed MoM revision surgery	15 (4.3)	12 (5.0)	6 (4.1)	6 (6.5)	1.86 (0.58-6.00)	.301
Revision or re-revision of MoM (%)						
Revision of primary MoM for ARMD	331 (95.7)	227 (95.0)	141 (95.9)	86 (93.5)	1.00 (ref)	
Re-revision (ie previous MoM revision surgery then developed ARMD)	15 (4.3)	12 (5.0)	6 (4.1)	6 (6.5)	1.64 (0.51-5.25)	.405
Primary implant type (%)						
Hip resurfacing	245 (70.8)	150 (62.8)	98 (66.7)	52 (56.5)	1.00 (ref)	
Total hip arthroplasty	101 (29.2)	89 (37.2)	49 (33.3)	40 (43.5)	1.54 (0.90-2.63)	.115
Primary implant design (%)						
BHR	154 (44.5)	104 (43.5)	64 (43.5)	40 (43.5)	1.00 (ref)	
Other	78 (22.5)	51 (21.3)	34 (23.1)	17 (18.5)	0.80 (0.40-1.62)	.534
Conserve	53 (15.3)	29 (12.1)	19 (12.9)	10 (10.9)	0.84 (0.36-1.99)	.696
Corail pinnacle	33 (9.5)	29 (12.1)	22 (15.0)	7 (7.6)	0.51 (0.20-1.30)	.158
Synergy BHR	28 (8.1)	26 (10.9)	8 (5.4)	18 (19.6)	3.60 (1.43-9.05)	**.006**
Primary implant head size (%)						
Less than 46 mm	101 (37.6)	80 (41.7)	52 (46.0)	28 (35.4)	1.00 (ref)	
46 mm	82 (30.5)	60 (31.3)	36 (31.9)	24 (30.4)	1.24 (0.62-2.47)	.545
Above 46 mm	86 (32.0)	52 (27.1)	25 (22.1)	27 (34.2)	2.01 (0.98-4.09)	.055
Symptoms (%)						
Local symptoms	323 (93.4)	221 (92.5)	133 (90.5)	88 (95.7)	2.32 (0.74-7.27)	.150
Systemic symptoms	2 (0.58)	2 (0.84)	1 (0.68)	1 (1.1)	1.60 (0.10-25.97)	.739
Blood metal ions						
Median cobalt in μg/l (IQR)	1.92 (0.65-7.50)	1.87 (0.65-8.02)	1.53 (0.29-7.20)	2.62 (0.88-9.40)	1.01 (0.99-1.02)	.418
Median chromium in μg/l (IQR)	3.38 (1.51-7.90)	3.51 (1.56-8.30)	3.21 (1.48-8.30)	3.93 (1.75-8.45)	1.00 (0.99-1.02)	.828
Radiograph						
Mean cup inclination in degrees (SD)	48.7 (10.8)	48.6 (10.8)	48.8 (10.7)	48.4 (11.2)	1.00 (0.97-1.02)	.773
Mean cup version in degrees (SD)	18.1 (10.4)	19.4 (10.4)	19.7 (11.1)	18.9 (9.3)	0.99 (0.97-1.02)	.594
Cup malposition (%)	183 (52.9)	135 (56.5)	86 (58.5)	49 (53.3)	0.81 (0.48-1.37)	.427
Stem/head malposition (%)	5 (1.5)	3 (1.3)	0 (0)	3 (3.3)	NA (no events in 1 group)	NA
Loose cup (%)	19 (5.5)	9 (3.8)	5 (3.4)	4 (4.4)	1.29 (0.34-4.94)	.709
Loose stem (%)	20 (5.8)	11 (4.6)	10 (6.8)	1 (1.1)	0.15 (0.02-1.20)	.073
Lysis cup (%)	125 (36.1)	101 (42.3)	65 (44.2)	36 (39.1)	0.81 (0.48-1.38)	.439
Lysis stem (%)	58 (16.8)	42 (17.6)	27 (18.4)	15 (16.3)	0.87 (0.43-1.73)	.684
Neck thinning (%)	49 (14.2)	34 (14.2)	24 (16.3)	10 (10.9)	0.63 (0.28-1.38)	.243
Impingement (%)	1 (0.29)	1 (0.42)	0 (0)	1 (1.1)	NA (only 1 event)	NA
Heterotopic ossification (%)	24 (6.9)	20 (8.4)	7 (4.8)	13 (14.1)	3.29 (1.26-8.60)	**.015**
Any cross-sectional imaging						
Any abnormality (% of those with imaging)	265 (84.9)	202 (84.5)	124 (84.4)	78 (84.8)	1.03 (0.50-2.13)	.929
Pseudotumors (PTs)						
PT numbers (%)	214 (68.6)	163 (68.2)	97 (66.0)	66 (71.7)	1.31 (0.74-2.31)	.353
PT consistency (% of all PT)						
Cystic	101 (48.1)	71 (44.1)	48 (50.0)	23 (35.4)	1.00 (ref)	
Mixed	97 (46.2)	83 (51.6)	46 (47.9)	37 (56.9)	1.68 (0.87-3.24)	.123
Solid	12 (5.7)	7 (4.4)	2 (2.1)	5 (7.7)	5.22 (0.94-28.95)	.059
PT location (% of all PT)						
Anterior ± lateral	88 (42.3)	64 (39.8)	33 (34.4)	31 (47.7)	1.00 (ref)	
Posterior ± lateral	61 (29.3)	48 (29.8)	33 (34.4)	15 (23.1)	0.48 (0.22-1.06)	.069
Anterior + posterior ± lateral	33 (15.9)	29 (18.0)	20 (20.8)	9 (13.9)	0.48 (0.19-1.21)	.120
Other	26 (12.5)	20 (12.4)	10 (10.4)	10 (15.4)	1.06 (0.39-2.91)	.903
Median PT volume in cm^3^ (IQR)	44.7 (14.0-117.2)	44.9 (13.4-130.0)	52.0 (14.4-166.4)	44.7 (13.3-82.8)	1.00 (0.99-1.01)	.920
Other image abnormalities						
Effusion	60 (19.2)	44 (18.4)	32 (21.8)	12 (13.0)	0.54 (0.26-1.11)	.094
Muscle atrophy/damage	24 (7.7)	17 (7.1)	9 (6.1)	8 (8.7)	1.46 (0.54-3.93)	.454
Tendon abnormality/damage	16 (5.1)	13 (5.4)	8 (5.4)	5 (5.4)	1.00 (0.32-3.15)	.998
Bursal distension/thickening	36 (11.5)	24 (10.0)	17 (11.6)	7 (7.6)	0.63 (0.25-1.58)	.325
**Intraoperative factors (%)**						
Median surgeon volume (range)	46 (1-70)	46 (1-70)	46 (3-70)	46 (1-70)	0.99 (0.98-1.01)	.460
Center						
Center 1	188 (54.3)	166 (69.5)	105 (71.4)	61 (66.3)	1.00 (ref)	
Center 2	158 (45.7)	73 (30.5)	42 (28.6)	31 (33.7)	1.27 (0.72-2.23)	.403
Posterior approach	253 (73.1)	189 (79.1)	125 (85.0)	64 (69.6)	0.40 (0.21-0.76)	**.005**
Intraoperative findings						
PT	189 (54.6)	117 (49.0)	75 (51.0)	42 (45.7)	0.81 (0.48-1.36)	.419
Effusion	122 (35.3)	93 (38.9)	57 (38.8)	36 (39.1)	1.02 (0.59-1.73)	.956
Soft-tissue damage	108 (31.2)	66 (27.6)	35 (23.8)	31 (33.7)	1.63 (0.91-2.89)	.098
Soft-tissue necrosis	25 (7.2)	12 (5.0)	4 (2.7)	8 (8.7)	3.40 (0.99-11.65)	.051
Cup malposition	82 (23.7)	58 (24.3)	33 (22.5)	25 (27.2)	1.29 (0.71-2.35)	.408
Stem/head malposition	16 (4.6)	11 (4.6)	6 (4.1)	5 (5.4)	1.35 (0.40-4.56)	.628
Loose cup	14 (4.1)	9 (3.8)	3 (2.0)	6 (6.5)	3.35 (0.82-13.74)	.093
Loose stem/head	24 (6.9)	10 (4.2)	8 (5.4)	2 (2.2)	0.39 (0.08-1.86)	.235
Lysis cup	184 (53.2)	136 (56.9)	85 (57.8)	51 (55.4)	0.91 (0.54-1.53)	.717
Lysis stem	44 (12.7)	30 (12.6)	19 (12.9)	11 (12.0)	0.91 (0.41-2.02)	.826
Infection	4 (1.2)	0 (0)	0 (0)	0 (0)	NA (no events in 1 group)	NA
Neck thinning	12 (3.5)	6 (2.5)	5 (3.4)	1 (1.1)	0.31 (0.04-2.71)	.291
Acetabular fracture	4 (1.2)	2 (0.84)	1 (0.68)	1 (1.1)	1.60 (0.10-25.97)	.739
Femoral fracture	8 (2.3)	3 (1.3)	3 (2.0)	0 (0)	NA (no events in 1 group)	NA
Metallosis	171 (49.4)	125 (52.3)	78 (53.1)	47 (51.1)	0.92 (0.55-1.56)	.766
Impingement	6 (1.7)	6 (2.5)	4 (2.7)	2 (2.2)	0.79 (0.14-4.43)	.793
Synovitis	18 (5.2)	16 (6.7)	8 (5.4)	8 (8.7)	1.65 (0.60-4.57)	.332
Revision performed						
All components	244 (70.5)	154 (64.4)	98 (66.7)	56 (60.9)	1.00 (ref)	
Single component (cup or stem/head)	74 (21.4)	57 (23.9)	28 (19.1)	29 (31.5)	1.81 (0.98-3.35)	.058
Modular only (head/liner)	28 (8.1)	28 (11.7)	21 (14.3)	7 (7.6)	0.58 (0.23-1.46)	.249
Type of implants						
Primary	230 (66.5)	152 (63.6)	93 (63.3)	59 (64.1)	1.04 (0.60-1.79)	.892
Revision	116 (33.5)	87 (36.4)	54 (36.7)	33 (35.9)	1.00 (ref)	
Revision head size						
Less than 36 mm	216 (62.4)	155 (64.9)	91 (61.9)	64 (69.6)	1.00 (ref)	
36 mm or above	130 (37.6)	84 (35.1)	56 (38.1)	28 (30.4)	0.71 (0.41-1.24)	.228
Cup fixation (% of all those cups revised)						
Cementless	295 (98.3)	201 (98.5)	119 (100)	82 (96.5)	1.00 (ref)	
Cemented	5 (1.7)	3 (1.5)	0 (0)	3 (3.5)	NA (no events in 1 group)	NA
Stem fixation (% of all those stems revised)						
Cementless	109 (41.6)	82 (50.9)	60 (57.1)	22 (39.3)	1.00 (ref)	
Cemented	153 (58.4)	79 (49.1)	45 (42.9)	34 (60.7)	2.06 (1.06-3.99)	**.032**
Revision bearing surface						
Ceramic-on-ceramic	78 (22.5)	46 (19.3)	23 (15.7)	23 (25.0)	1.00 (ref)	
Metal-on-polyethylene	107 (30.9)	54 (22.6)	37 (25.2)	17 (18.5)	0.46 (0.20-1.04)	.061
Ceramic-on-polyethylene	80 (23.1)	70 (29.3)	47 (32.0)	23 (25.0)	0.49 (0.23-1.05)	.067
Oxinium-on-polyethylene	78 (22.5)	69 (28.9)	40 (27.2)	29 (31.5)	0.73 (0.34-1.53)	.401
Metal-on-metal	3 (0.87)	0 (0)	0 (0)	0 (0)	NA	NA
Bone graft (acetabular ± femoral)	118 (34.1)	88 (36.8)	59 (40.1)	29 (31.5)	0.69 (0.40-1.19)	.180

Statistically significant *P* values (<0.05) highlighted in bold text.

ARMD, adverse reactions to metal debris; BHR, Birmingham Hip Resurfacing; CI, confidence interval; IQR, interquartile range; LR, logistic regression; NA, not available; SD, standard deviation.

### Outcomes

**Fig. 1:**
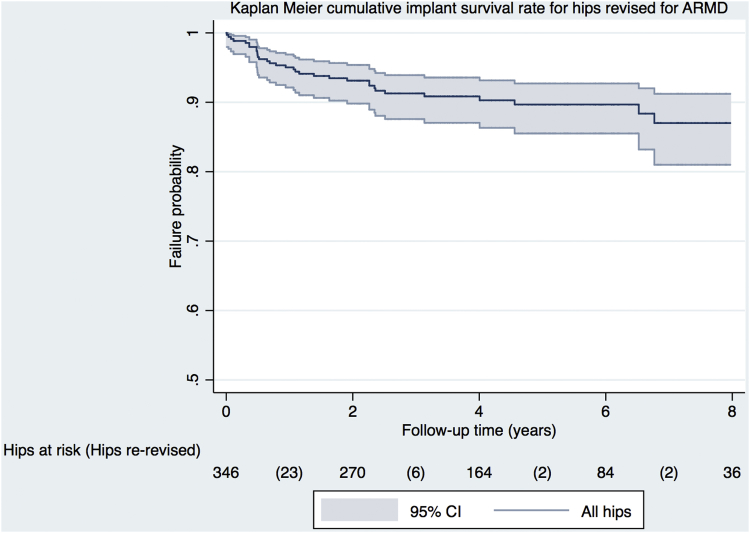
Kaplan-Meier cumulative implant survival rate following revision surgery performed for adverse reactions to metal debris (ARMD). The shaded area represents the respective upper and lower limits of the 95% confidence intervals (CIs). Risk table indicates the number of hips at risk at 2-year intervals, with the corresponding number in brackets detailing the number of hips undergoing re-revision surgery during each 2-year interval.

Intraoperative complications (all femoral or acetabular fractures) occurred in 1.5% (n = 5). One or more postoperative complications occurred in 17.6% of hips (n = 61), which included re-revision surgery (n = 33), further surgery excluding re-revision (n = 28), and complications not requiring surgery (n = 9). Re-revision surgery was performed at a mean of 1.58 years from ARMD revision (range = 0.01-6.77 years), with the commonest indications being dislocation (n = 13; 39.4% of all re-revisions), ARMD recurrence (n = 5; 15.2%), and aseptic acetabular component loosening (n = 5; 15.2%). Death occurred in 2.6% (n = 9) of patients following ARMD revision (range = 0.07-8.09 years), with one death occurring within 90 days of surgery. Mean follow-up time for non–re-revised patients was 4.75 years from revision (range = 1.0-16.0 years). The cumulative implant survival rate 7 years after ARMD revision was 87.0% (CI = 81.0%-91.2%; 60 hips at risk) (Figure 1).

The commonest reasons for further surgery excluding re-revision were closed reductions for dislocation (n = 19; 67.9% of all further surgery) and washout/debridement for infection/hematoma (n = 4; 14.3%). The commonest complications not requiring surgery were superficial wound infections (n = 4) and femoral nerve palsy (n = 2). All adverse events are summarized (Table 2).

**Table 2:** Intraoperative and Postoperative Adverse Outcomes Following ARMD Revision Surgery.

Outcome of Interest	Number of Hips	Complication Details
Intraoperative complications (5 in total)	1	Greater trochanter fracture (no treatment)
	1	Calcar crack (treated with wires)
	1	Femoral shaft fracture (treated with cables)
	1	Acetabular fracture (no treatment)
	1	Femoral shaft fracture (treated with open reduction and internal fixation)
Re-revision surgery (33 in total)	13	Dislocation
	5	ARMD recurrence (2 associated with dislocation and/or fracture)
	5	Aseptic acetabular component loosening
	3	Infection
	3	Periprosthetic femoral fracture
	4	Other (1 each of below):
		Acetabular component malposition
		Reclean of inflamed trochanteric bursa and change of modular components
		Implant fracture through modular neck
		Stem malalignment + leg length discrepancy
Further surgery not including re-revision (28 in total)	19	Closed reduction for dislocation under anesthesia
	4	Washout/debridement for wound infection or hematoma
	2	Abductor repair/reattachment
	1	Excision of pseudotumor recurrence (no implants changed)
	1	Psoas release for irritation (arthroscopic)
	1	Stenting of stenotic external iliac artery (claudication)
Other complications not needing any surgery (9 in total)	4	Superficial wound infection (treated with antibiotics only)
	2	Femoral nerve palsy (1 transient/1 permanent)
	1	Leg length discrepancy (1.5 cm) with neuropathic foot
	1	Deep vein thrombosis
	1	Cellulitis (treated with antibiotics only)

ARMD, adverse reactions to metal debris.

In patients not undergoing re-revision, the median OHS was 36 (interquartile range = 25-45). Poor PROMs were observed in 22.8% (n = 79). Thirty-nine percent (n = 135) of all patients had a poor outcome following ARMD revision. Of hips with poor outcomes, 117 fulfilled 1 criterion for a poor outcome, 17 fulfilled 2 criteria, and 1 hip fulfilled 3 criteria.

### Risk Factors for Poor Outcomes

Predictors of poor outcomes were assessed in 239 patients (69% of cohort) who all had blood metal ions and cross-sectional imaging prerevision (poor outcome observed in 38.5%; n = 92).

In the univariable analysis, preoperative predictors of a poor outcome included shorter time (under 4 years) from primary to revision surgery, revision of a specific metal-on-metal total hip arthroplasty design (Synergy BHR), and radiographic evidence of heterotopic ossification (Table 1). Blood metal ions did not predict poor outcomes. In patients with imaging pseudotumors, the consistency, location, and volume were not associated with poor outcomes. In the univariable analysis, intraoperative predictors of a poor outcome were surgical approaches other than posterior and cemented stem fixation (vs cementless) (Table 1).

### Predictive Models for Poor Outcomes

A multivariable model (AUC = 68.4%) involving only preoperative factors identified one statistically significant predictor of a poor outcome, namely shorter time (under 4 years) from primary to revision surgery (odds ratio = 2.12; CI = 1.00-4.46; *P* = .049) (Table 3).

**Table 3:** Preoperative Predictors of Poor Outcomes Following ARMD Revision Surgery (Multivariable Logistic Regression Model).

Preoperative Factor (AUC = 68.4%)	Odds Ratio (95% CI)	*P* Value
Primary surgery factors		
Time to ARMD revision under 4 y	2.12 (1.00-4.46)	**.049**
Other diagnoses for native hip eg, dysplasia (vs primary osteoarthritis)	1.81 (0.91-3.62)	.093
Failed primary MoM hip revised to another MoM hip (vs primary osteoarthritis)	2.52 (0.68-9.38)	.169
Imaging factors		
Heterotopic ossification on radiograph	2.52 (0.89-7.16)	.082
Loose stem on radiograph	0.17 (0.02-1.42)	.101
Bursal distension on imaging	0.43 (0.13-1.44)	.172
Effusion on imaging	0.56 (0.26-1.25)	.158
Muscle atrophy on imaging	3.43 (0.92-12.75)	.065

Statistically significant *P*-values (<0.05) highlighted in bold text.

AUC, area under the curve; CI, confidence interval; ARMD, adverse reactions to metal debris.

A multivariable model (AUC = 74.0%) involving intraoperative factors identified a number of variables which significantly influenced outcomes (Table 4). Single-component revisions (acetabular or femoral; vs all-component revisions: OR = 2.99, CI = 1.50-5.97) and loose acetabular components at revision (OR = 4.66, CI = 1.04-20.92) increased the risk of poor outcomes. Intraoperative factors reducing the risk of poor outcomes included the posterior surgical approach (OR = 0.22, CI = 0.10-0.49), revision head sizes of 36 mm and above (vs under 36 mm: OR = 0.37, CI = 0.18-0.77), ceramic-on-polyethylene revision bearings (OR vs ceramic-on-ceramic = 0.30, CI = 0.14-0.66), and metal-on-polyethylene revision bearings (OR vs ceramic-on-ceramic = 0.37, CI = 0.17-0.83).

**Table 4:** Intraoperative Predictors of Poor Outcomes Following ARMD Revision Surgery (Multivariable Logistic Regression Model).

Intraoperative Factor (AUC = 74.0%)	Odds Ratio (95% CI)	*P* Value
Surgeon volume of revision ARMD cases	0.99 (0.98-1.01)	.104
Posterior approach for revision (vs other approach)	0.22 (0.10-0.49)	**<.001**
Intraoperative findings		
Soft-tissue damage at revision	1.62 (0.83-3.18)	.158
Loose acetabular component at revision	4.66 (1.04-20.92)	**.045**
Lysis acetabular component at revision	1.77 (0.81-3.85)	.153
Pseudotumor at revision	0.52 (0.27-1.01)	.054
Reconstruction performed		
Single-component (acetabular or femoral) revision (vs all component revision)	2.99 (1.50-5.97)	**.002**
36 mm or larger revision head (vs less than 36 mm)	0.37 (0.18-0.77)	**.007**
Ceramic-on-polyethylene revision bearing (vs ceramic-on-ceramic)	0.30 (0.14-0.66)	**.003**
Metal-on-polyethylene revision bearing (vs ceramic-on-ceramic)	0.37 (0.17-0.83)	**.016**
Use of bone graft (acetabular ± femoral)	0.46 (0.20-1.05)	.064

Statistically significant *P* values (<0.05) highlighted in bold text.

AUC, area under the curve; CI, confidence interval.

In patients undergoing ARMD revision where intraoperative modifiable factors were optimized (ie posterior approach, all components revised, and 36 mm or larger ceramic-on-polyethylene or metal-on-polyethylene bearings used), the risk of a poor outcome was 10% compared with 40% in patients undergoing reconstruction with other strategies.

## Discussion

Although there is some evidence that outcomes following ARMD revision have improved since the initial poor short-term outcomes [9], [10], the studies available are small single-center cohorts with short-term follow-up [8]. We have studied the largest nonregistry cohort to date, with the 2-center and multisurgeon design improving the generalizability of our findings to similar centers undertaking ARMD revisions. Our large cohort with mid-term follow-up therefore provides a comprehensive appraisal of the outcomes following ARMD revision, which can be used to informatively counsel MoMHA patients prerevision about their likely prognosis.

The risk of intraoperative complications and mortality was reassuringly low and similar to registry data [19]. Previous studies have reported implant survival rates of 88%-90% at 3 years to 5 years following ARMD revision [17], [18], [19]. Our implant survival rate at 7 years (87.0%) is therefore similar to these previous studies and also comparable with the 7 year implant survival observed in registries after non-MoM revisions for conventional modes of failure (85%-87% depending on fixation and articulation) [5]. The commonest re-revision indications reported here (dislocation, ARMD recurrence, and aseptic acetabular loosening) are consistent with the literature, with the reasons why ARMD revisions are prone to these modes of failure described previously [8], [19].

Although it is reassuring that the catastrophic short-term implant failure rates following ARMD revision [9], [10] are no longer observed, it is important to consider other outcomes of interest including PROMs and postoperative complications. These end points have not been appraised consistently in previous studies and are not available in registries [8]. Many patients (39%) experienced a poor outcome following ARMD revision despite having surgery at specialist centers by experienced surgeons. The most common reasons for this were poor PROMs, re-revision surgery, and further surgery excluding re-revision (namely closed reductions for dislocation). The high risk of a suboptimal outcome following ARMD revision despite specialist management highlights the importance of detailed prerevision counseling so patients are fully aware of the likely outcomes.

Surgeons currently have difficulty when managing MoMHA patients with ARMD with regards to the threshold for revision and type of reconstruction. This relates to a lack of evidence, with previous studies being underpowered when assessing predictors of a poor outcome [7], [15], [17], [18]. Unsurprisingly worldwide regulatory authorities provide variable recommendations about the revision threshold [16]. Surgeons therefore largely rely on expert opinion (level 5 evidence) to manage patients [39], [40], [41], which itself has been shown to be problematic [42]. We believe that our study is the first appropriately powered cohort which attempts to identify thresholds for revision surgery in MoMHA patients with ARMD using important preoperative factors.

The only preoperative factor significantly predicting a poor outcome was shorter time (under 4 years) from primary to revision surgery. The overall clinical performance of the preoperative threshold model was therefore poor. Recent data from the National Joint Registry for England and Wales also observed that a shorter time between primary MoMHA and revision for ARMD was associated with an increased risk of re-revision surgery [19]. We believe the short time between primary and revision procedures is likely to reflect the most aggressive and earliest ARMD cases where patient, surgical, and implant factors were all markedly suboptimal for undergoing MoMHA. These patients typically presented before we understood the entity of ARMD with large destructive lesions requiring early revision, but unfortunately, they experienced poor results after revision surgery [9], [15], [43]. Over time, our understanding of ARMD improved, patients underwent regular surveillance (including blood metal ions and imaging), and the threshold for offering revision surgery was gradually lowered [9], [13], [14], [15]. Patients revised for ARMD over more recent years have therefore had lower blood metal ions and less severe/destructive imaging, which would account for the majority of cases we studied. We believe that this may explain why important preoperative factors (such as ions and imaging) were not found to be predictors of poor outcomes in the present study and that the short-time between primary and revision was the only preoperative predictor which encompassed the early and most aggressive ARMD cases. However, given the rare use of MoMHA for a number of years, the widespread awareness of ARMD, and regular surveillance of MoMHA patients, it is suspected this short time between primary and revision is no longer relevant for future patients developing ARMD [5], [6], [13], [14].

Blood metal ion concentrations, features on cross-sectional imaging (including pseudotumor volume/consistency and muscle damage) and radiographs (including acetabular component position and osteolysis) did not predict poor outcomes following ARMD revision, although this is contrary to some much smaller studies with shorter follow-up [7], [15], [17], [18]. Our data therefore suggest that no robust thresholds exist for recommending revision in MoMHA patients with evidence of ARMD.

The performance of the model based on intraoperative factors was good, and the model contained numerous modifiable factors therefore suggesting that surgeons can influence the outcomes following ARMD revisions. The only nonmodifiable factor was a loose acetabular component at revision, which had a 4.7 fold increased risk of poor outcomes. Patients with this poor prognostic feature should be appropriately counseled postrevision and should undergo more regular surveillance to monitor osseointegration of the revision construct given that acetabular component loosening requiring re-revision has been commonly observed after ARMD revision and appears to be a complex problem to manage in the presence of ARMD [8], [43]. At the time of ARMD revision, it is also recommended that surgeons do more than normal to ensure secure acetabular fixation to facilitate osseointegration, such as by using more screws and/or by using highly porous implant surfaces. We found that no other intraoperative findings, including pseudotumor, soft-tissue necrosis, and osteolysis, were significantly associated with poor outcomes.

Modifiable risk factors of poor outcomes included surgical approach, and the type of revision preformed (including femoral head size and articulation). Using a posterior approach at revision was associated with a 78% reduced risk of poor outcomes. Elective arthroplasty data suggest that the posterior approach is associated with better PROMs and lower short-term mortality compared with the anterolateral approach [44], [45]. The posterior approach is considered more muscle sparing than the anterolateral approach. Problems therefore seen more commonly following the anterolateral approach include nerve injury [46], reduced muscle strength [47], and limping [48]. These problems invariably influence patient mobility and PROMs. It is recognized that a surgeon’s choice of approach may be limited by the anatomical location of ARMD and/or the approach used for the primary procedure. However, surgeons should attempt to use the posterior approach where possible for ARMD revisions.

Single-component (acetabular or femoral) revisions were associated with a 3-fold increased risk of poor outcomes compared with all component revisions. Although some authors have achieved promising results with this strategy when revising MoMHAs for ARMD [11], most studies support our findings, even in non-MoMHAs revised for ARMD [10], [19], [29], [49]. Although single-component revisions have advantages, such as reducing the time and potential morbidity of removing well-fixed components, it is possible they are being overused, such as in cases where positioning of the retained component may not be absolutely optimal [8], [19]. We believe there is now good evidence that clinical outcomes are inferior in single-component MoMHA revisions for ARMD compared with revising all components, even in stemmed MoMHAs. Surgeons wishing to use single-component revisions in selected cases must ensure they appropriately counsel patients before revision regarding the likely prognosis.

Revision femoral head sizes of 36 mm and above had a 63% reduced risk of poor outcomes following ARMD revision compared with smaller sizes. Ceramic-on-polyethylene and metal-on-polyethylene revision bearings had a 70% and 63% reduced risk of poor outcomes, respectively, compared with ceramic-on-ceramic revision bearings. Dislocation is a major problem following ARMD revision observed here and previously [8]. Hip stability is compromised in these cases by destruction and/or resection of affected soft-tissues, and reducing the large diameter MoM bearing to a smaller non-MoM articulation. Recent registry data also observed that ceramic-on-ceramic bearings used for ARMD revisions were associated with higher re-revision rates, with ceramic-on-polyethylene performing best [19]. The reasons why ceramic-on-ceramic bearings have inferior outcomes to hard-on-soft articulations remains unclear; however, we consider there is now good evidence that large-diameter (36 mm or above) hard-on-soft articulations (preferably ceramic-on-polyethylene bearings) should be used for ARMD revisions.

This study had recognized limitations. Its retrospective nature introduces potential bias regarding the preoperative (namely cross-sectional imaging reports) and intraoperative (namely revision operation records) data collected. We were therefore limited by assessing only the data recorded in these reports, which in some cases did not categorically confirm or refute the presence of all the abnormalities we have presented. Although these issues have been considered previously [28], it is clear that a similar prospective study would take many years to complete; therefore, our work provides the best available evidence in the interim. It is recognized that change in PROMs may have been more useful for our poor outcome definition rather than postrevision PROMs; however, we did not have data on prerevision PROMs to calculate this. Owing to the studies large nature, we were unable to perform detailed appraisal and grading of the histopathological specimens [25], [26] to determine how these features correlated with outcomes, which may have provided useful information for our models. Similarly, we were unable to review all postrevision radiographs and perform routine postrevision blood metal ions, which would have both provided important information on patients who may require future re-revision surgery. Although we used comprehensive methods to determine outcomes in all patients, it is possible some patients may have undergone further surgery which we were unaware of. Finally, our statistical models were based on a subgroup of patients undergoing complete preoperative investigation (ions and imaging), which may decrease the power of our models. This was an inevitable limitation given the study was retrospective and that the diagnosis and investigation of patients with ARMD has evolved over time [9], [15].

## Conclusions

This large cohort study demonstrated 39% of patients experience poor outcomes following MoMHA revision for ARMD. This information will allow surgeons to informatively counsel patients prerevision about the expected prognosis. No robust threshold exists for recommending ARMD revision; therefore, surgeons must continue to make decisions on an individual case basis. Patients undergoing early revisions (within four-years of primary) and those with loose acetabular components at revision should be counseled about potentially experiencing poor outcomes. However our work does suggest that surgeons can make intraoperative decisions that influence the outcomes following ARMD revision. We therefore recommend that the best outcomes following MoMHA revision for ARMD can be achieved if surgeons use the posterior approach, revise all MoMHA components, and use large-diameter (36 mm or above) ceramic-on-polyethylene or metal-on-polyethylene articulations.

## Appendix A Supplementary Data

Conflict of Interest Statement for Matharu

Conflict of Interest Statement for Berryman

Conflict of Interest Statement for Dunlop

Conflict of Interest Statement for Revell

Conflict of Interest Statement for Judge

Conflict of Interest Statement for Murray

Conflict of Interest Statement for Pandit
