# Supplementary material for: No Threshold Exists for Recommending Revision Surgery in Metal-on-Metal Hip Arthroplasty Patients With Adverse Reactions to Metal Debris: A Retrospective Cohort Study of 346 Revisions
Source: J Arthroplasty. 2019 Jul;34(7):1483–91. doi: 10.1016/j.arth.2019.03.022 (PMC6590389; doi:10.1016/j.arth.2019.03.022)
Supplement: Conflict of Interest Statement for Matharu [file mmc1.doc]

# CONFLICT OF INTEREST STATEMENT

***The Journal of Arthroplasty***

(Adopted from the American Academy of Orthopaedic Surgeons disclosure statement)

The following form **must be filled out completely and submitted by each author (example, 6 authors, 6 forms). If no discloser is required, please write/type “none” at the end of each sentence.**

Manuscript Title: - Can we identify thresholds for revision surgery in metal-on-metal hip arthroplasty patients with adverse reactions to metal debris? A retrospective cohort study of 346 revisions

1. Royalties from a company or supplier (The following conflicts were disclosed)

None

2. Speakers bureau/paid presentations for a company or supplier (The following conflicts were disclosed)

None

3A. Paid employee for a company or supplier (The following conflicts were disclosed)

None

3B. Paid consultant for a company or supplier (The following conflicts were disclosed)

Medicolegal work for Leigh Day

3C. Unpaid consultants for a company or supplier (The following conflicts were disclosed)

None

4. Stock or stock options in a company or supplier (The following conflicts were disclosed)

None

5. Research support from a company or supplier as a Principal Investigator (The following conflicts were disclosed)

Surgical Research Fellowship awarded from The Royal College of Surgeons of England.

Clinical Research Fellowship awarded from Arthritis Research UK.

Research Grants from The Orthopaedics Trust, and The Royal Orthopaedic Hospital Hip Fund.

6. Other financial or material support from a company or supplier (The following conflicts were disclosed)

None

7. Royalties, financial or material support from publishers (The following conflicts were disclosed)

None

8. Medical/Orthopaedic publications editorial/governing board (The following conflicts were disclosed)

None

9. Board member/committee appointments for a society (The following conflicts were disclosed)

None

**Each author must sign AND print or type his/her name, date and submit a separate form**

In addition, one BLINDED Conflict of Interest form (no author names used) should be submitted per manuscript with all author disclosures.

Gulraj Matharu Gulraj Matharu 12^th^ February 2019

Author Name (Print or Type) Author Signature Date
