# Supplementary material for: No Threshold Exists for Recommending Revision Surgery in Metal-on-Metal Hip Arthroplasty Patients With Adverse Reactions to Metal Debris: A Retrospective Cohort Study of 346 Revisions
Source: J Arthroplasty. 2019 Jul;34(7):1483–91. doi: 10.1016/j.arth.2019.03.022 (PMC6590389; doi:10.1016/j.arth.2019.03.022)
Supplement: Conflict of Interest Statement for Judge [file mmc5.doc]

# CONFLICT OF INTEREST STATEMENT

***The Journal of Arthroplasty***

(Adopted from the American Academy of Orthopaedic Surgeons disclosure statement)

The following form **must be filled out completely and submitted by each author (example, 6 authors, 6 forms). If no discloser is required, please write/type “none” at the end of each sentence.**

Manuscript Title: - Can we identify thresholds for revision surgery in metal-on-metal hip arthroplasty patients with adverse reactions to metal debris? A retrospective cohort study of 346 revisions

1. Royalties from a company or supplier (The following conflicts were disclosed)

NONE

2. Speakers bureau/paid presentations for a company or supplier (The following conflicts were disclosed)

NONE

3A. Paid employee for a company or supplier (The following conflicts were disclosed)

NONE

3B. Paid consultant for a company or supplier (The following conflicts were disclosed)

Servier, UK Renal Registry, Oxford Craniofacial Unit, IDIAP Jordi Gol, Freshfields Bruckhaus Deringer.

3C. Unpaid consultants for a company or supplier (The following conflicts were disclosed)

NONE

4. Stock or stock options in a company or supplier (The following conflicts were disclosed)

NONE

5. Research support from a company or supplier as a Principal Investigator (The following conflicts were disclosed)

Consortium research grants from ROCHE

6. Other financial or material support from a company or supplier (The following conflicts were disclosed)

NONE

7. Royalties, financial or material support from publishers (The following conflicts were disclosed)

NONE

8. Medical/Orthopaedic publications editorial/governing board (The following conflicts were disclosed)

A member of the Data Safety and Monitoring Board (which involved receipt of fees) from Anthera Pharmaceuticals, INC.

9. Board member/committee appointments for a society (The following conflicts were disclosed)

NONE

**Each author must sign AND print or type his/her name, date and submit a separate form**

In addition, one BLINDED Conflict of Interest form (no author names used) should be submitted per manuscript with all author disclosures.

ANDREW JUDGE ANDREW JUDGE 12^th^ February 2019

Author Name (Print or Type) Author Signature Date
